# Structural differences in the semantic networks of younger and older adults

**DOI:** 10.1038/s41598-022-11698-4

**Published:** 2022-12-12

**Authors:** Dirk U. Wulff, Thomas T. Hills, Rui Mata

**Affiliations:** 1 Department of Psychology, University of Basel, Missionsstrasse 60-62, 4055 Basel, Switzerland; 2 Max Planck Institute for Human Development, Berlin, Germany; 3 University of Warwick, Coventry, England

**Keywords:** Human behaviour, Cognitive ageing, Learning and memory

## Abstract

Cognitive science invokes semantic networks to explain diverse phenomena, from memory retrieval to creativity. Research in these areas often assumes a single underlying semantic network that is shared across individuals. Yet, recent evidence suggests that content, size, and connectivity of semantic networks are experience-dependent, implying sizable individual and age-related differences. Here, we investigate individual and age differences in the semantic networks of younger and older adults by deriving semantic networks from both fluency and similarity rating tasks. Crucially, we use a megastudy approach to obtain thousands of similarity ratings per individual to allow us to capture the characteristics of individual semantic networks. We find that older adults possess lexical networks with smaller average degree and longer path lengths relative to those of younger adults, with older adults showing less interindividual agreement and thus more unique lexical representations relative to younger adults. Furthermore, this approach shows that individual and age differences are not evenly distributed but, rather, are related to weakly connected, peripheral parts of the networks. All in all, these results reveal the interindividual differences in both the content and the structure of semantic networks that may accumulate across the life span as a function of idiosyncratic experiences.

## Introduction

Semantic networks are a form of knowledge representation that represent relations between items, such as concepts, using a graph-type system consisting of nodes and their interconnections. Such networks have been postulated as the representational basis of our cognitive system^1–3^ and are, therefore, an integral part of prominent models of memory^4^, reasoning^5^, and creativity^6,7^. Past work has often made the simplifying assumption that a common semantic network can be used to understand human semantic cognition^4,5,8–11^. This assumptions is implicit, for instance, in efforts to model retrieval from memory^12^, judgments of relatedness^13^, or decision making^14^ using large-scale word vector spaces and free-association networks. However, general theories of learning and development^15,16^, as well as empirical findings^17–19^, suggest that semantic networks could vary considerably between individuals and across the life span. Crucially, researchers now seem to agree that understanding experience-dependent changes and individual variation in cognition is an important frontier for the science of aging^20^.

Aging research has made significant progress in the past decades in quantifying age-related changes in semantic cognition, including large increases in the size of the knowledge-store across adult development, perhaps best documented in the large differences in vocabulary size across the adult life span^21,22^. More recently, however, research suggests that individual learning and life span development can also lead to changes in the structure of human knowledge^23–25^. For example, recent efforts have used data from large-scale free-association studies to show that older adults’ semantic networks are less connected, efficient, and structured relative to those of younger adults^12,18^.

Quantifying individual and age differences in the size and structure of human knowledge is important because this can help identify the sources underlying cognitive changes in later life. Older adults tend to perform worse on a broad set of cognitive tasks, and such findings are commonly attributed to a decline in fluid cognitive abilities^26,27^. However, changes in the size and structure of the knowledge representation can masquerade as changes in fluid cognitive abilities, which may be sometimes difficult to disentangle^9^. Moreover, many have argued that changes in the underlying size and structure of representations contributes directly to age differences in cognitive performance, for example, due to activation-spreading across many targets in memory (fan effect^28^) or difficulties in discrimination learning between many similar items^16^. Consequently, it has been proposed that it is important to understand the links between the size and structure of human knowledge and individual and age differences in fluid abilities (e.g.,^25^).

**Figure 1:**
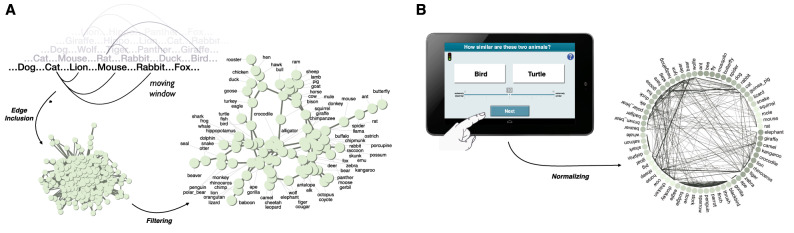
Methodological approach. Panel (**A**) illustrates the two steps, edge inclusion and filtering, involved in inferring networks from semantic fluency sequences. For details see Materials and Methods. The resulting network is based on 142 sequences of the older adults’ group of study 1. To simplify the visualization more conservative inferences settings were employed than used in the analyses reported below. Panel (**B**) illustrates the creation of networks from similarity ratings by normalizing individuals’ responses to the range of 0 and 1. The weighted network is based on the average ratings of the older adults’ group of study 3.

One first step needed to understand the contribution of semantic networks to age differences in cognitive performance is to document the changes in the size and structure of semantic networks across the life span. The few existing studies comparing the semantic networks of younger and older adults have used different elicitation methods to extract only aggregate-level networks of younger and older adults, while also focusing on different parts of the semantic network. In the present study, we seek to address the shortcomings of existing efforts to document potential life span differences in the structure of semantic networks in several ways (see Fig. 1).

First, we investigate age differences in the size and semantic network structure for aggregates of younger and older groups obtained from a semantic fluency task (e.g., “Name all animals you can”). Semantic fluency tasks are typically employed to measure fluid cognitive abilities, for instance, in screening instruments for age-related cognitive pathology (e.g., Alzheimer’s disease^29^). We study age differences in this paradigm under different conditions: Our analyses of the semantic structure of fluency productions are the first to include age comparisons using different semantic categories (animal vs. country) and different retrieval time allowances (1 minute vs. 10 minutes). Little is known about how structural properties vary across different areas of the semantic network^30,31^. Nonetheless, retrieval speed represents an established difference between younger and older adults’ cognition^26^. Consequently, by comparing networks from semantic fluency across different time conditions, we will be able to assess the robustness of past findings with respect to semantic content and situations where older adults may catch-up or even outperform younger adults in semantic fluency when given the opportunity to search their potentially larger semantic stores. Also, past work suggests that younger and older adults differ across semantic categories^32^, so comparing the network structure of different categories further contributes to understanding the factors that drive such differences.

Second, we adopt a megastudy approach^33^ to provide the first comparison of younger and older adults’ semantic networks at the level of individuals. This involves collecting over 2,000 similarity ratings from each participant. This is crucial for two reasons. Aggregate networks likely do not accurately reflect the structure of individual level networks^9,12,34^. Further, similarity ratings likely recruit different retrieval processes than those underlying the two elicitation methods past studies have relied on, semantic fluency and free associations. Consequently, this will allow us to rule out the possibility that age differences in semantic networks are driven by aggregation bias or retrieval processes associated with a particular elicitation method. Furthermore, we will relate individual differences in the structure of similarity rating networks to individual differences in education and cognitive performance, and thereby assess supposed drivers and consequences of semantic network structure^12^.

In sum, we aim to contribute to mapping the structural differences in the semantic networks of younger and older adults by assessing whether they are robust across different semantic domains (i.e., animals, countries), different retrieval processes (e.g., fluency, similarity rating), and levels of analysis (i.e., aggregate vs. individual) so as to shed light on age differences in semantic network structure.

## Results

### Age-related differences in fluency networks

Semantic fluency is a neuropsychological test that requires participants to retrieve as many elements as possible from a natural category^35^, say, animals, within in a given amount of time. Research has begun to analyze semantic fluency data in novel ways to extract from them semantic networks^36^ and understand individual and age differences in semantic cognition^23,37^. Such approaches leverage the fact that the proximity of elements within the sequence of responses should reveal information on whether two elements are connected in an underlying semantic network. Several algorithms utilizing this principle have been proposed. To infer semantic networks of younger and older adults, we rely on a random walk plus filtering algorithm, which was recently found to predict human behavior better than other algorithms^37^. In the first step, this algorithm adds to a single network for each age group edges for every pair of elements that occurred less than two positions apart from each other across all semantic fluency sequences of the age group. Then, in a second step, all edges in the network that were added only once across all sequences or were less frequent than expectation derived from random behavior are removed (see Figure 1A). Previous research has found this approach to produce plausible networks that predict human behavior better than other network inference methods for fluency data^37,38^.

**Figure 2:**
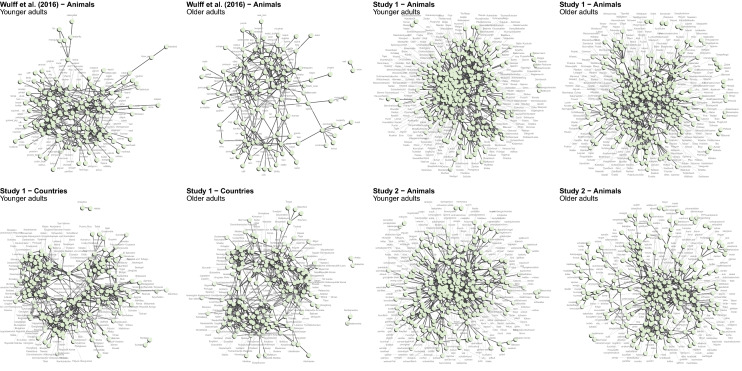
Fluency networks. The figure shows the networks estimated for younger and older adults in each of the four data sets analyzed. Labels are not displayed on top of their nodes so as not to obscure the structural characteristics of the network. The figures suggest similarity in the semantic networks of the two age groups, with clustering of semantic-related items in close proximity. For details on the network inference mechanism, see Methods.

We compared semantic networks of younger and older adults on the basis of four semantic fluency data sets, stemming from published work^39^ and two new studies (see Methods for details). Table 1 provides an overview of the data sets. Following previous work^18,39,40^, we compared younger and older adults’ networks with respect to three macroscopic network measures: average degree (connectivity, $\langle k \rangle$), average local clustering coefficient (structuredness, *C*), and average shortest path length (efficiency, *L*). These metrics are frequently employed to characterize the structure of cognitive networks and have been successfully linked to various measures of cognitive performance (for reviews, see^11,25,41^): For instance, degree has been linked to speed of retrieving words in lexical decison tasks^42^, clustering has been linked to retrieval success in cued recall tasks^43^, and shortest path length has been linked to faster information processing^44^. To avoid any confounding influences of network size and content, younger and older adults’ networks were compared on the largest connected, common subgraph, containing only words that were produced by both younger and older adults. Figure 2 shows the networks estimated for younger and older adults in each of the four data sets analyzed. Overall, the figures suggest similar semantic relations between items, showing that the network inference mechanism generates plausible, intuitive semantic networks. These figures do not allow, however, an easy and direct comparison of network characteristics across groups, which is typically done by relying on quantitative indices of macroscopic network characteristics, such as connectivity indices (e.g., degree, shortest path length; cf.^18,45^).

**Figure 3:**
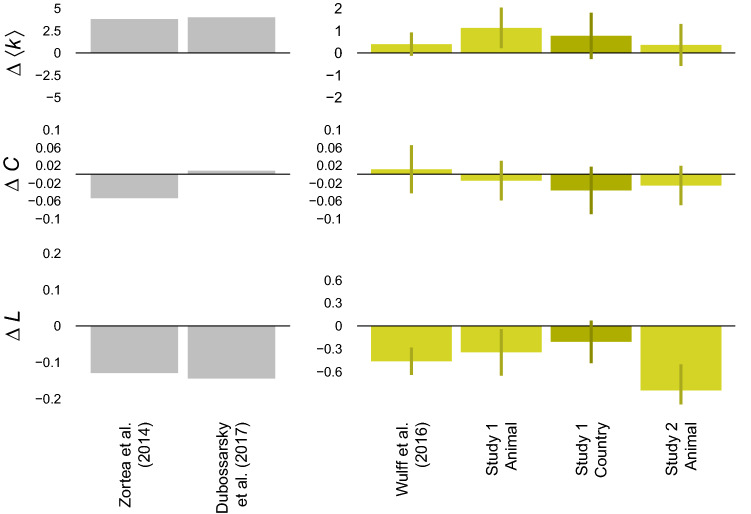
Differences in the macroscopic structure of younger and older adults’ fluency networks. Gray bars show the difference between the younger and older adults’ age group in Zortea et al.^40^ and that of age 30 and 70 in Dubossarsky et al.^18^, respectively. Yellow bars show differences in networks inferred from the four fluency data sets. Error bars show 95% bootstrapped confidence intervals. Note: $\Delta \langle k \rangle$-Differences in average degrees; $\Delta C$-Difference in average clustering coefficients; $\Delta L$-Difference in average shortest path lengths.

We present a comparison of network indices in Fig. 3. Compared to older adults, the networks of younger adults showed consistently higher average degrees and lower average shortest path lengths. These differences were found to be reliable for one (Study 2—Animals) and three (all but study 2—Countries) data sets according to bootstrap tests (see Supplementary Material). However, results for the average clustering coefficient were mixed. A multiverse analysis^46^ evaluating the results under various implementations of our inference method suggests that inference is robust for degree and shortest path length, but not for clustering, supporting the differences found for degree and shortest path length and providing an explanation for the mixed results in the latter (see Supplementary Material). These results corroborate the existence of systematic structural differences between younger and older adults’ semantic networks in terms of connectivity and efficiency, but not clustering.

Two additional findings concerning younger and older adults’ semantic fluency data are worth noting. First, in the two studies that gave participants 10 minutes to retrieve items from semantic memory, there were no differences in the number of items produced by younger and older adults, as determined by permutation tests (Table 1). Compared with the shorter retrieval periods of previous studies (cf.^47–49^), the longer retrieval period of 10 minutes seems to eliminate older adults’ disadvantage of slower memory retrieval. Second, as a group, older adults produced more unique category items across all four data sets, as measured by the number of unique items relative to the number of responses produced by an age group (cf. $\frac{u}{\Sigma n}$ in Table 1), as determined by a permutation test (Table 1), which is supportive of the notion that older adults possess a larger mental lexicon than younger adults^21^. Despite such differences, the age-related patterns in macroscopic network structure generalize across the different domains and conditions, which speaks to the generality of these findings across elicitation procedures (cf.^12,18^).

**Table 1:** An Overview of Fluency Data and their Inferred Macroscopic Network Structure.

Data set	Age (Range)	N	t	$\bar{n}$	$\frac{u}{\Sigma n}$
Wulff et al. (2016)	52.5 (29-65)	142	1 min	21.2$^*$	.09$^*$
	73.7 (66-94)	142	1 min	17.9$^*$	.11$^*$
Study 1 (Animal)	25 (18-34)	41	10 min	90.7	.15$^a$
	71.1 (66-81)	71	10 min	89.6	.18$^*$
Study 1 (Country)	25 (18-34)	41	10 min	75.3	.08$^*$
	71.1 (66-81)	71	10 min	69.7	.11$^*$
Study 2 (Animal)	24 (18-32)	36	10 min	92.6	.17
	70 (65-78)	36	10 min	88.6	.19

* Found to be significantly different between younger and older adults according to permutation tests; N = number of participants; t = time limit of the fluency task; $\bar{n}$ = average number of non-duplicate, valid responses per individual; $\frac{u}{\Sigma n}$ = number of unique responses *u* divided by the sum of number responses *n* for all individuals in the group.

### Age-related differences in individual-level similarity networks

A potential criticism of extant comparisons of younger and older adults’ networks is that they lump together the data of many individuals to form aggregate networks, thus obscuring individual differences. To address this limitation, we conducted a comparison of younger and older adults’ semantic networks at the level of the individual. Specifically, we elicited a large number of similarity ratings and constructed networks directly from each individual’s responses. Aside from avoiding problems of aggregation, this approach had five additional advantages: First, similarity ratings likely recruit different memory retrieval processes and may overall be less affected by such processes than semantic fluency, permitting an independent and, potentially, cleaner assessment of network structure. Second, by requiring participants to rate a common set of words, similarity ratings likely are less affected by vocabulary differences between younger and older adults. Third, similarity ratings deliver direct estimates of the connection strength between words, sidestepping the need to infer edges using complex algorithms. Fourth, similarity ratings deliver graded responses permitting the construction of networks with weighted edges. Finally, because network statistics are available for each individual, the comparison between younger and older adults’ networks can be carried out using standard methods of statistical inference.

**Figure 4:**
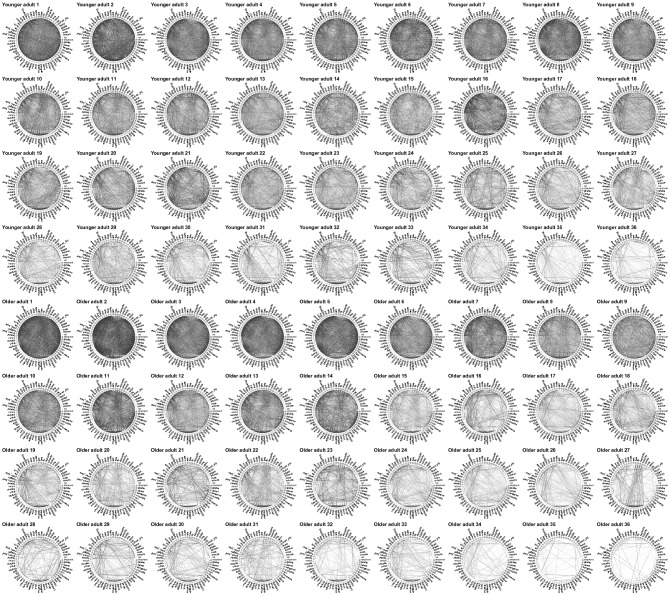
Similarity rating networks. Each individual plot shows the network of one individual under $w_{min}=.1$. The first four  rows show the networks of younger adults, the bottom four rows those of older adults. Please note that networks are ordered by network strength to facilitate a visual comparison of between- and within-group variability in network structure. Edges’ weights have been scaled according to $w^2$ to increase visibility. Nodes are ordered and colored according to ten animal categories. These are, starting at $0^\circ$, African animals (plus kangaroo), large apes, birds, farm animals, fish, forest animals, pets, reptiles, and rodents. Animals names were translated from German.

In our study, each of 36 younger and 36 older participants provided a total of 2,253 similarity ratings, of which 1,953 were given to all possible pairs of 63 common animals and the remaining 300 to a set of repeat pairs, for which we found reliability to be high (older adults: $r = .76$, younger adults: $r = .74$). Participants were instructed to rate similarity based on the animal’s degree of relatedness or association. We constructed networks by, first, mapping an individual’s ratings from the original scale of 1 (extremely dissimilar) to 20 (extremely similar) to the scale of 0 to 1, by setting a person’s minimum and maximum rating to 0 and 1, respectively. This was done for two reasons: to account for differences in scale use and to introduce a 0 indicating the absence of edges. Second, we placed edges between all 63 animal nodes with weights equal to the transformed ratings. Finally, we eliminated edges with weights below a threshold $w_{min} = [0, .1, .2, .3, .4]$. This last step was necessary to be able to determine the average local clustering coefficient, which is not defined for completely connected networks, while also providing us with a means to assess the robustness of our results to the choice of threshold. Figure 4 shows the 72 networks obtained from younger and older adults under $w_{min}=.1$.

**Figure 5:**
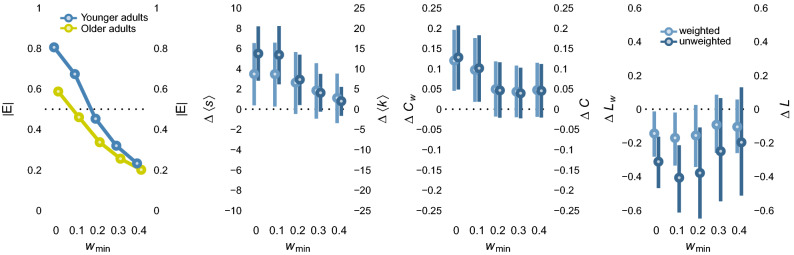
Differences in the macroscopic structure of younger and older adults’ similarity rating networks. Blue and yellow circles, in panel 1, correspond to younger and older adults, respectively. In panels 2 to 4, light blue circles and dark blue circles correspond to differences between the younger and older adults’ networks derived from weighted and unweighted networks, respectively. Error bars show 95% bootstrapped confidence intervals. Note: |*E*| - Proportion of edges relative to fully-connected graph; $\Delta \langle s \rangle$, $\Delta \langle k \rangle$ - Differences in average strengths/degrees (unweighted); $\Delta C_w$, $\Delta C$ - Difference in average clustering coefficients of weighted/unweighted networks; $\Delta L_w$, $\Delta L$ - Difference in average shortest path lengths of weighted/unweighted networks.

Across all values of $w_{min}$, compared to older adults, the networks of younger adults showed consistently higher average degrees ($\langle k \rangle$) and lower average shortest path lengths (*L*), and also higher local clustering coefficients (*C*) (see Fig. 5). We found the same pattern of results when the networks were analyzed as unweighted networks. For small values of $w_{min}$, where more than 50% of all edges were retained, i.e., $w_{min} \in (0, .1)$, moderate to large effects were observed, of which many were reliably distinct from 0 as indicated by the 95% confidence intervals. Effects for more restrictive values of $w_{min}$, i.e., $w_{min} > .1$ pointed in the same direction, but they were smaller in size and, due to larger variance, were mostly not reliably different from 0. These results corroborate the structural differences found for aggregate networks and demonstrate, for the first time, systematic age-related differences in the structure of semantic networks at the level of the individual obtained from a megastudy using semantic similarity ratings.

Moreover, analyses reported in the Supplementary Material confirm the existence of aggregation biases. For the average degree, the clustering coefficient, and the average shortest path length, but not the average strength, estimates based on aggregate networks, which we derived by averaging networks within age groups, were considerably higher than the majority of estimates for individual-level networks. Aggregate networks, however, still revealed group differences consistent with those observed on the individual level, suggesting some level of robustness of group comparisons obtained from aggregate data.

### Locating age-related differences in semantic network structure

Past work on the development of semantic knowledge suggests that cumulative linguistic experience and general learning process combine to create specific semantic structures that allow efficient discrimination learning^16^. Crucially, that work proposes that such learning processes involve the strengthening of some associations while weakening others to allow differentiating between meaningful and meaningless pairs of items in memory. One important consequence of this process is that age differences in network structure may not be homogeneous across pairs of associations due to the interaction of learning and cumulative experience.

**Figure 6:**
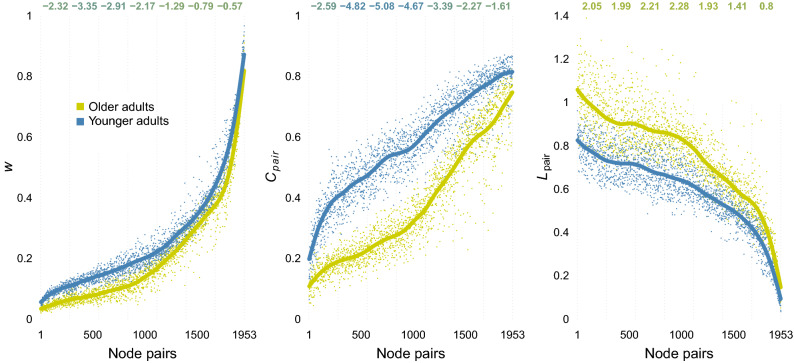
Comparisons between younger and older adults’ networks across all 1,953 node pairs. The panels show separately for younger (blue) and older (yellow) adults the average edge weights under $w_{min} = 0$ (left panel), the proportion of triangles that existing edges form with other edges under $w_{min} = .1$ (middle panel), and the shortest paths between the nodes $w_{min} = .1$ (bottom panel). The numbers on top of each panel show the Cohen’s *d* (younger–older adults) for bins of 200 node pairs. Note: *w*—Edge weight; $C_{pair}$—Proportion of triangles formed by pair; $L_{pair}$—Distance between between nodes in pair.

To shed light on the differences between younger and older adults’ networks, we compared their networks on the level of node pairs with respect to three metrics that directly underlie the macroscopic results in Fig. 5 and allow us to assess homogeneity of age differences across node pairs. Specifically, for each of the 1,953 node pairs, we compare the edge weight *w* under $w_{min} = 0$ (corresponding to $\langle s \rangle$ and $\langle k \rangle$), the proportion with which the pair forms triangles with other nodes ($C_{pair}$) under $w_{min} = .1$, and the path length connecting the pair ($L_{pair}$) also under $w_{min} = .1$. Figure 6 displays these results separately for younger and older adults with node pairs ordered by the average edge weight *w* across both age groups. Ordering edges in this way allows direct inference-by-eye to reveal whether age-differences emerge uniformly across the network.

We observed consistent differences between younger and older adults in terms of all three metrics. Specifically, the edge weights and the proportion of triangles were consistently lower for older than younger adults, whereas path lengths were consistently larger. Crucially, we observed that the differences between older and younger adults were considerably larger for the lower half of node pairs. Thus, the differences between younger and older adults’ networks appear to be mainly due to peripheral regions in the network, where edge weights are small, triangles rare, and shortest path lengths long.

We should note that the results above do not seem to be explained by age differences in use of the scale. We observed the judgments of younger and older adults not to differ in terms of the judged minimum ($d = 0, 95\%CI = [-.46, 46]$) or the judged maximum ($d = .26, 95\%CI = [-.2, 72]$). However, we did find younger and older adults to differ in terms of the ratings‘ average ($d = .56, 95\%CI = [.09, 1.03]$) and, crucially, the ratings’ skewness ($d = -.51, 95\%CI = [-.98, -.04]$), with older adults’ ratings being lower on average and more right skewed. This suggests that younger and older adults interpreted and used the end points of the scales in the same way, and differed only in how they distributed the word pairs in between the end points, as would be expected from different perceptions of similarity between judged pairs.

### Assessing the links between cumulative experience, network structure and cognitive performance

**Figure 7:**
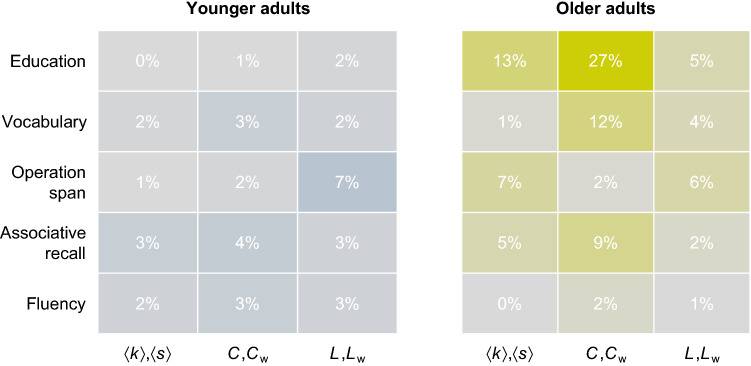
Shared variances between network structure, education, and cognitive performance. Numbers display the average proportion of shared variance across $w_{min} \in [0,.1,.2,.3,.4]$ and weighted and unweighted versions of individuals’ networks. Note: $\langle s \rangle$, $\langle k \rangle$—Differences in average strengths/degrees (unweighted); *C*, $C_w$—Difference in average clustering coefficients of weighted/unweighted networks; *L*, $L_w$—Difference in average shortest path lengths of weighted/unweighted networks.

The assessment of individual differences in semantic networks gives us the opportunity to assess their links to proposed drivers and consequences of semantic network structure (see,^12^). If, as past work has suggested, cumulative experience is responsible for structural differences in semantic memory, then differences in the level of education experienced by individuals should be related to semantic network structure. The analysis presented in Fig. 7 reveals that this link was present for older adults, but not for younger adults. Specifically, we observed that a dummy variable coding whether a person received a college education shared between 5% (shortest path length) and 27% (clustering) of variance with the structure of older adults’ networks, whereas there was practically no shared variance between education and younger adults’ networks (see Fig. 7). These results suggest that it is not education by itself, but possibly other forms of life experience that impact individuals’ semantic networks. Consistent with this assessment, results reported in the Supplementary Material show that the effects of age group on network structure are not fully accounted for by education level (or gender). Another corollary of the idea that cumulative experience drives network structure is that older adults differ more from each other as a function of their different accumulated experiences^25^. We tested this expectation in two ways. First, we compared the within-group variance of network measures and observed that those of older individuals was between 55% (degree) and 116% larger than those of younger adults. Second, we evaluated within age-group agreement in terms of edge weights. Specifically, we compared all pairs of individual networks using a weighted Jaccard index (*JI*). We found older adults’ networks to be considerably less similar to each other ($\overline{JI}=.33$) than younger adults’ networks ($\overline{JI}=.45$; $d=.97$). Overall, these results are compatible with the idea that cumulative exposure to linguistic and other information contributes to individual differences in the structure of semantic networks.

Past work suggests that the structure of semantic networks may drive cognitive performance, however, this link has so far not been assessed from the perspective of individual differences. To fill this gap, we calculated the shared variance between network structure and vocabulary size, working memory capacity assessed using the operation span task, the number of correct decisions in an associative recall task, and the number of retrievals in the animal fluency task. The results presented in Fig. 7 reveal two important insights. First, network structure and cognitive performance shared, on average, more variance for older than for younger adults, likely due to the larger differences between the network structures of older adults. Second, network structure and cognitive performance shared relatively little variance (median = 3%), with the exception of maybe the links between clustering and vocabulary (12% shared variance) and between clustering and associative recall (9%) among older adults. Overall, these results provide only weak support for the idea that semantic network structure drives individual differences in cognitive performance.

## Discussion

We investigated differences in the networks of younger and older adults at both the group and the individual level. Our group-level analyses using semantic fluency data replicate previously observed differences between networks of younger and older adults (e.g.,^18,23,40^): The aggregate younger adults’ networks based on verbal fluency exhibited larger average degrees and lower average shortest path lengths than older adults’ networks, but the networks of the two age groups did not systematically differ in their average clustering coefficients. Overall, these results indicate that semantic networks may become increasingly sparse with age, with the connectivity between items decreasing with age. Importantly, we extend past work by showing that these age patterns generalize across categories (animals, countries) and time constraints (1 vs. 10 minutes), suggesting that such age-related differences are not a function of specific elicitation choices and generalize across domains.

In addition, analyses of individual networks estimated from a similarity-judgment task involving thousands of judgments from the same individuals ruled out potential problems of aggregation and confirmed the differences in average degrees and lower average shortest path lengths, while additionally revealing systematic differences in terms of average clustering coefficients, in the direction of lower clustering in older adults’ semantic networks. We found age differences were especially pronounced for weakly-related, peripheral regions of the network. Further, older adults’ networks were more variable and considerably less similar to each other than younger adults’ networks, and this variability covaried with level of education. All in all, these results provide converging evidence that the semantic networks of younger and older adults differ systematically not only in content, as has been amply suggested in past work^21^, but also in their structure^25^. Our results are congruent with the idea of a progressively idiosyncratic nature of semantic representations across the life span, leading to more distinct semantic representations between individuals over time. Further, our findings are novel in suggesting that individual and age differences may be strongest for peripheral parts of semantic representations, which emphasize the importance of investigating a large swath of individuals’ semantic representations to understand the environmental and cognitive contributions to individual differences in semantic cognition.

We should point out a number of limitations in our work. First and foremost, we must acknowledge that we cannot definitively determine to what extent the age differences described above are due to age differences in representation and/or control processes involved in searching and selecting information from memory. The type of network models we adopt here to describe lexical associations are, in principle, compatible with mechanistic explanations based on both representation and process and, therefore, cannot fully arbitrate between the two^50^. Our finding that results generalize across elicitation conditions (time constraints), domains (animals, countries), and tasks (semantic fluency, similarity judgement) could be indicative of age differences being due to differences in the underlying representation, but only to the extent that one can confidently assume different processes of search and comparison across the different conditions, domains, and tasks. It seems plausible that the underlying cognitive processes are perhaps not identical but at least similar, as all share aspects of controlled selection, involving the activation of concepts (e.g., “animal”) and their features (e.g., “has wings”). There are two main approaches that could be interesting to further address the role of representation and process in engendering age differences in the semantic networks estimated from lexical tasks. One approach could involve additional independent measures to statistically account for the contribution of control processes using an individual differences approach (e.g.,^51^). Another approach involves making use of neuroimaging techniques to directly measure mechanisms of control and memory retrieval. Past work suggests that representation and semantic control rely on distinct (but interacting) brain regions^52^ and this information could potentially be leveraged to provide an estimate of the role of control processes in semantic cognition.

Second, we are unable to derive strong conclusions about how the differences in semantic networks impact younger and older adults’ cognitive performance. The three properties of semantic network structure considered—size, connectedness, efficiency, and structuredness—have been linked empirically and theoretically to cognitive performance (for reviews see^11,12,25^), but in most cases these links were established at the level of words or word pairs, rather than individuals’ overall performance (but see^31^). For instance, research has found that words with higher degrees (connectedness) are more likely to be retrieved in semantic fluency or episodic memory tasks (e.g.,^12^). Furthermore, while it is plausible that word-level effects translate to differences in overall performance, we observed only relatively weak links between cognitive network structure and performance. One possible explanation is the lack of semantic overlap between the networks’ contents and the cognitive tasks we deployed. Alternatively, the lack of strong links between network structure and cognitive performance could underpin the possibility that the observed differences in semantic network structure reflect differences in representation, rather than fluid cognitive ability. Indeed, this was one of the motivations behind using similarity ratings as opposed to deriving networks from free association or fluency data alone. The latter approaches might confound fluid abilities and memory representation more strongly than similarity ratings^9,12^.

Third, on a related note, we do not detail a specific mechanism to account for the interaction between cumulative experience and network structure. Consequently, a key challenge for future research lies in developing models for the age-related changes in the structure of semantic networks reported here. One promising proposal stems from models of discriminative learning, whereby increasing experience leads weakly and strongly related contents in memory to be driven further apart from each other, resulting in a topological expansion of the network. The nature of structural differences, the observations of amplified differences for more weakly related words, as well as the lower similarity between older adults’ compared to younger adults’ networks, seem to support this notion. However, so far, discriminative learning has only been successfully employed to account for age differences in paired-associate learning^15,16^. Whether such a mechanism can be expanded to account for the full set of results presented here remains an open question.

Fourth, and finally, our work made use of an extreme-group comparison design by comparing groups of younger relative to older adults. This type of design is not optimal to study the role of cumulative experience that is thought to underlie age differences in the content and structure of lexical and semantic networks. Ideally, estimates of cumulative experience and associated semantic networks would be obtained longitudinally for large samples of individuals and across long spans of time involving years or decades. One major difficulty with such studies will be mapping semantic networks for specific individuals but such efforts are under way^12^.

Despite its limitations, our work has some important implications for understanding and modeling human cognition. In many tasks that are presumed to be linked to the structure of semantic networks, older adults are known to perform worse than younger adults^26^, which is often considered a consequence of declining fluid abilities^26,27^. Our and similar findings of systematic differences in semantic networks open up an alternative route leading to age differences in cognitive performance, whereby older adults’ cognitive performance shows apparent decline because of the consequences of learning for the size and the structure of semantic networks. In turn, our finding that age differences may be particularly pronounced in weakly connected, peripheral parts of semantic networks could have implications for future tests of theories of individual and age differences in semantic cognition that may, or may not, make predictions concerning different parts of semantic representations.

Our results may have implications beyond our theoretical understanding of healthy cognitive aging. Lacking a cure, the best way to battle the “dementia epidemic” is timely diagnosis and early treatment^53,54^. The diagnosis of mild cognitive impairments and early dementia is, however, still predominately based on tests of cognitive performance^53^. Instruments such as the short dementia screener DemTect^29^ or the neuropsychological battery CERAD^55^ involve an individual undergoing a series of standard cognitive tasks, including several of the tasks listed above. Understanding the role of age-related changes in the structure of semantic networks promises to improve our interpretation of current instruments for dementia screening and diagnosis. Further research in this direction could lead to more personalized instruments that can detect changes in cognitive performance earlier and with higher sensitivity by focusing on specific parts of semantic representations than is currently done.

In sum, we have presented converging results from semantic fluency and similarity judgment tasks concerning structural differences in the semantic networks of younger and older adults. Older adults seem to possess richer more idiosyncratic networks, characterized by smaller average degree and longer path lengths relative to those of younger adults. Our results emphasize the importance of considering how life span cognitive development and cumulative experience shape the content and structure of individuals’ semantic cognition.

## Methods

### Fluency data

Four data sets from three studies were used to infer networks from fluency data. The first data set was obtained from^39^, who jointly analyzed the data of two published studies, i.e., from Hills et al.^49^ and the Midlife in the United States (MIDUS3) longitudinal study. The data of Hills et al.^49^ contain a one-minute animal fluency task collected at Stanford University, CA, of a total of 201 participants aged 27 to 99 (Mdn = 68). The MIDUS3 data contained one-minute animal fluency data—recorded in phone interviews—from 104 individuals aged 34 to 83 (Mdn = 59). Audio recordings were transcribed by us (see Supplementary Material). Following Wulff et al.^39^, we eliminated 21 individuals with fewer than 10 fluency productions and mini-mental state values lower than 26, which is indicative of either low attention to the task or the onset of age-related disorders, before combining the two data sets for a total sample of 282 individuals. Groups of younger and older adults were created by splitting the data at the median age. This resulted in groups of 142 individuals each aged 29 to 65 years old and 66 to 94 years old, respectively. The original data of our first study were collected in the context of another investigation into age-difference in decision making running in the laboratories of the Max Planck Institute (MPI) for Human Development, Berlin. We collected 10-minute fluency data for both animals and countries from 71 older adults and 41 younger adults. Responses were recorded using a microphone and transcribed by us. Participants were recruited through the internal participant database of the MPI for Human Development. The older adults’ age ranged from 65 to 80 years with a median age of 70 years, the younger adults’ age ranged from 17 to 33 with a median age of 25. Participants were paid 10€/hour for participation. The second study was also conducted at the Max Planck Institute for Human Development using participants from the MPI’s internal database. We collected 10-minute fluency data for animals from 36 older adults and 36 younger adults. Responses were recorded using a microphone and transcribed by us. The older adults’ age ranged from 65 to 78 years with a median age of 70 years, the younger adults’ age ranged from 18 to 32 with a median age of 24. Participants in study 3 also completed measures of vocabulary size, working memory capacity (i.e., operation span task), and associative recall (see Supplementary Material). Participants were paid 10€/hour for participation. Study 1, 2 and 3 were approved by the internal review board of the Max Planck Institute for Human Development. All studies were performed in accordance with relevant guidelines and regulations. Participants provided informed consent at the beginning of each study.

Fluency data were subjected to minimal preprocessing. Responses were scrutinized for category membership and spelling. A lenient criterion was used to assess category membership to retain as much of the original data as possible. In the case of animals, all nonfictional entries that described entire, nonhuman, and nonfictional animals were retained. This led us to exclude a few cases from the data, such as Godzilla, cat eye, or animal trainer. Similarly, in the case of countries, we retained all existing and named territories such as Istrien, a region of Italy, Croatia and Slovenia, the desert Sahara or cities, but not nonexistent, fictional territories such as Middle-earth. Spelling was hand-corrected on the basis of the Merriam-Webster online dictionary. Overall 96.8–99% of responses were retained in the analysis.

### Measures of macroscopic network structure

The average degree of a network $G = (V, E)$, with nodes (or vertices) *V* and edges *E*, is defined as $\langle k \rangle =\frac{2|E|}{|V|}$ for unweighted networks and as $\langle k \rangle =\frac{2}{|V|(|V|-1)}\sum _{i,j \in V; i \ne j} a_{ij}w_{ij}$, where $a_{i,j}$ denotes the presence of an edge between nodes *i* and *j* and $w_{i,j}$ the according edge weight. The average degree or strength, as it is commonly referred to for weighted networks, describes the average connectivity in the network. The average local clustering coefficient for unweighted networks is defined as $C = \frac{1}{|V|}\sum _{i \in V} C_{i}$ with $C_{i} = \frac{2}{|k_i|(k_i-1)} \sum _{j,h \in N_i}{a_{jh}}$ and $k_i$ being the degree of node *i* and $N_i$ the set of neighbors to *i*. For weighted networks, $C_{i}^{w} = \frac{1}{|s_i|(k_i-1)} \sum _{j,h \in N_i}{\frac{w_{ij}+w_{ih}}{2}a_{ij}a_{ih}a_{jh}}$ with $s_i = \sum _{j \in N_i}{w_{j}}$ being the strength of node *i*, the weighted analog to $k_i$. The local clustering coefficient describes the degree of transitivity in the network and is related to network modularity^56^. It is often conceived of as an indicator of the structuredness of a network^57^. The average shortest path length is defined as $L = \frac{2}{|V|(|V|-1)}\sum _{i,j \in V; i \ne j} L_{ij}$ where $L_{ij}$ is the length of shortest path between nodes *i* and *j*, also known as the geodesic distance. For weighted networks, $L_{ij}$ is the sum of weights rather than the length. The average shortest path length describes the average distance between nodes. Low average shortest path lengths have been associated with efficient information processes^44,58^.

### Network inference approach

Networks were inferred from semantic fluency data based on the community model developed by Goñi et al.^38^ and studied by Zemla and Austerweil^37^. The model is based on a two-step procedure. First, nodes and edges are included for every pair of responses that occurred within a distance of *l* responses. For instance, for the response sequence “dog, cat, mouse, rabbit” and a criterion of $l = 2$, edges would be included for all pairs less than three responses apart, excluding only the pair dog and rabbit, which are three responses apart. Second, an edge is identified as a true edge if the frequency of the connected words occurring with *l* or fewer steps apart exceeded a frequency threshold $t_{min}$ reflecting the required minimum frequency of co-occurring within *l* responses to be considered in the first place, as well as a frequency threshold $t_{chance}$. The latter is derived from the probability $p_{ij}^{linked}$ of two words occurring within *l* responses by chance, which is calculated as $p_{ij}^{linked} = p_ij^{co-occur}*p_{ij}^{\ge l}$. Furthermore, $p_{ij}^{co-occur}$, the probability of two words to co-occur within a fluency sequence, and $p_{ij}^{\ge l}$, the probability that two responses are no more than *l* responses apart, are calculated as $p_{ij}^{co-occur} = \frac{f_{i}f_{j}}{MM}$ and $p_{ij}^{\ge l} = \frac{2}{N(N-1)} (-lN \frac{l(l+1)}{2})$ with $f_i$, $f_j$ denoting the number of times two responses occur across *M* sequence and *N* denotes the average number of productions per sequence. $t_{chance}$ is then defined as the $1-\alpha$ quantile of the binomial distribution $B(M,p_{ij}^{linked})$. Consistent with prior literature, we set $l = 2$, $t_{min} = 1$, and $\alpha = .05$^37,38^ for our main analyses. In addition, we evaluate the robustness of the results in a multiverse analysis^46^ presented in the Supplementary Material.

### Similarity ratings

Similarity ratings were collected in the context of study 3 and prior to participants completing the semantic fluency task. Participants took home a tablet to provide, over the course of roughly one week, on a scale from 1 to 20, similarity ratings for 2,268 pairs of animals, consisting of each possible pair of 63 frequently occurring animals and 315 repeated pairs. The 63 animals were selected on the basis of the semantic fluency responses of study 2 in a manner that equated word frequency across younger and older adult age groups. See Supplementary Material. Reliability was found to be high in both younger and older adults with correlations of $r = .76$, $r = .74$ for younger and older adults, respectively. Participants were paid 10€/hour for participation in the lab session and a flat fee of 44.1€ for providing the similarity ratings.

### Statistical comparisons

Group comparisons of semantic fluency statistics were carried out using permutation and bootstrap tests. Group differences in the number of retrievals and the number of unique retrievals were tested by comparing them against a null distribution of 10,000 samples generated by randomly assigning individuals to our two age groups. Group differences in fluency network measures were tested by generating sampling distribution consisting of 10,000 samples per group by drawing from the groups’ fluency sequences with replacement. To account for the differences in group sizes in the two data sets of study 1, all study 1 analyses are on repeated random samples of matching group sizes. Group comparisons of similarity network statistics were also carried using bootstrap tests based on 10,000 bootstrap samples. Wherever possible we report the results as 95% confidence intervals rather than p-values to account for the non-confirmatory nature of our investigation.

## Supplementary Information

Supplementary Information.

## References

[1] Baronchelli A, Ferrer-i-Cancho R, Pastor-Satorras R, Chater N, Christiansen MH (2013). Networks in cognitive science. Trends Cogn. Sci..

[2] Beer RD (2000). Dynamical approaches to cognitive science. Trends Cogn. Sci..

[3] Borge-Holthoefer J, Arenas A (2010). Semantic networks: Structure and dynamics. Entropy.

[4] Anderson JR (1983). A spreading activation theory of memory. J. Verbal Learn. Verbal Behav..

[5] Collins AM, Loftus EF (1975). A spreadingactivation theory of semantic processing. Psychol. Rev..

[6] Kenett, Y. N. *et al.* Flexibility of thought in high creative individuals represented by percolation analysis. *Proceedings of the National Academy of Sciences*, 201717362 (2018). 10.1073/pnas.1717362115 PMC5798367 29339514

[7] Beaty, R. E. *et al.* Robust prediction of individual creative ability from brain functional connectivity. *Proceedings of the National Academy of Sciences*, 201713532 (2018). 10.1073/pnas.1713532115 PMC5798342 29339474

[8] Hills TT, Jones MN, Todd PM (2012). Optimal foraging in semantic memory. Psychol. Rev..

[9] Hills, T. T. & Kenett, Y. N. Is the mind a network? Maps, vehicles, and skyhooks in cognitive network science. *Topics in Cognitive Science* (2021). 10.1111/tops.12570 34435461

[10] Jones MN, Hills TT, Todd PM (2015). Hidden processes in structural representations: A reply to abbott, austerweil, and griffiths (2015). Psychol. Rev..

[11] Siew, C. S., Wulff, D. U., Beckage, N. M. & Kenett, Y. N. Cognitive network science: A review of research on cognition through the lens of network representations, processes, and dynamics. *Complexity*, 2108423 (2019).

[12] Wulff, D. U., De Deyne, S., Aeschbach, S., & Mata, R. (2021). Understanding the aging lexicon by linking individuals’ experience, semantic networks, and cognitive performance. *PsyArXiv*. 10.1111/tops.12586 PMC9303352 35040557

[13] Kraemer, P. M., Wulff, D. U., & Gluth, S. (2021). A sequential sampling account of semantic relatedness decisions. *PsyArXiv*.

[14] Bhatia S (2019). Predicting risk perception: New insights from data science. Manage. Sci..

[15] Ramscar M, Hendrix P, Shaoul C, Milin P, Baayen H (2014). The myth of cognitive decline: Non-linear dynamics of lifelong learning. Top. Cogn. Sci..

[16] Ramscar M, Sun CC, Hendrix P, Baayen H (2017). The mismeasurement of mind: Life-span changes in paired-associate-learning scores reflect the “cost” of learning, not cognitive decline. Psychol. Sci..

[17] Benedek M, Kenett YN, Umdasch K, Anaki D, Faust M, Neubauer AC (2017). How semantic memory structure and intelligence contribute to creative thought: A network science approach. Think. Reason..

[18] Dubossarsky H, De Deyne S, Hills TT (2017). Quantifying the structure of free association networks across the life span. Dev. Psychol..

[19] Morais AS, Olsson H, Schooler LJ (2013). Mapping the structure of semantic memory. Cogn. Sci..

[20] Lindenberger U (2014). Human cognitive aging: Corriger la fortune?. Science.

[21] Verhaeghen P (2003). Aging and vocabulary scores: A meta-analysis. Psychol. Aging.

[22] Keuleers E, Stevens M, Mandera P, Brysbaert M (2015). Word knowledge in the crowd: Measuring vocabulary size and word prevalence in a massive online experiment. Q. J. Exp. Psychol..

[23] Cosgrove AL, Kenett YN, Beaty RE, Diaz MT (2021). Quantifying flexibility in thought: The resiliency of semantic networks differs across the lifespan. Cognition.

[24] Nation, K. Nurturing a lexical legacy: Reading experience is critical for the development of word reading skill. *NPJ Sci. Learn.* **2**(3) (2017). 10.1038/s41539-017-0004-7 PMC6220205 30631450

[25] Wulff DU, De Deyne S, Jones MN, Mata R (2019). The aging lexicon consortium. New perspectives on the aging lexicon. Trends Cognit. Sci..

[26] Salthouse TA (2010). Selective review of cognitive aging. J. Int. Neuropsychol. Soc..

[27] Healey MK, Kahana MJ (2016). A four-component model of age-related memory change. Psychol. Rev..

[28] Buchler NE, Reder LM (2007). Modeling age-related memory deficits: A two-parameter solution. Psychol. Aging.

[29] Kalbe E, Kessler J, Calabrese P, Smith R, Passmore A, Brand Ma, Bullock R (2004). Demtect: A new, sensitive cognitive screening test to support the diagnosis of mild cognitive impairment and early dementia. Int. J. Geriatr. Psychiatry.

[30] Luce PA, Pisoni DB (1998). Recognizing spoken words: The neighborhood activation model. Ear Hear..

[31] Howard DV (1983). A multidimensional scaling analysis of aging and the semantic structure of animal names. Exp. Aging Res..

[32] Verheyen S, Droeshout E, Storms G (2019). Age- related degree and criteria differences in semantic categorization. J. Cogn..

[33] Keuleers E, Balota DA (2018). Megastudies, crowdsourcing, and large datasets in psycholinguistics: An overview of recent developments. Q. J. Exp. Psychol..

[34] Wulff, D. U., Aeschbach, S., De Deyne, S., & Mata, R. (in press). Data from the myswow proof-of-concept study: Linking individual semantic networks and cognitive performance. Journal of Open Psychology Data.

[35] Bousfield WA (1953). The occurrence of clustering in the recall of randomly arranged associates. J. Gen. Psychol..

[36] Henry JD, Crawford JR, Phillips LH (2004). Verbal fluency performance in dementia of the alzheimer’s type: A meta-analysis. Neuropsychologia.

[37] Zemla, J. C. & Austerweil, J. L. Estimating semantic networks of groups and individuals from fluency data. *Computational Brain & Behavior*, 1–23 (2018). 10.1007/s42113-018-0003-7 PMC6555428 31179436

[38] Goñi J, Arrondo G, Sepulcre J, Martincorena I, de Mendizábal NV, Corominas-Murtra B, Bejarano B, Ardanza-Trevijano S, Peraita H, Wall DP (2011). The semantic organization of the animal category: Evidence from semantic verbal fluency and network theory. Cogn. Process..

[39] Wulff, D. U., Hills, T. T., Lachman, M., & Mata, R. (2016). The aging lexicon: Differences in the semantic networks of younger and older adults. *Proceedings of the 38th Annual Conference of the Cognitive Science Society*. Austin, TX, 907-912.

[40] Zortea M, Menegola B, Villavicencio A, Salles JFd (2014). Graph analysis of semantic word association among children, adults, and the elderly. Psicologia: Reflexão e Crítica.

[41] Kenett, Y. N., Beckage, N. M., Siew, C. S. & Wulff, D. U. Cognitive network science: A new frontier. *Complexity*, 6870278 (2020).

[42] De Deyne S, Navarro DJ, Storms G (2013). Better explanations of lexical and semantic cognition using networks derived from continued rather than single-word associations. Behav. Res. Methods.

[43] Nelson DL, Bennett DJ, Gee NR, Schreiber TA, McKinney VM (1993). Implicit memory: Effects of network size and interconnectivity on cued recall. J. Exp. Psychol. Learn. Mem. Cogn..

[44] Bullmore E, Sporns O (2012). The economy of brain network organization. Nat. Rev. Neurosci..

[45] Steyvers M, Tenenbaum J (2005). The large-scale structure of semantic networks: Statistical analyses and a model of semantic growth. Cogn. Sci..

[46] Steegen S, Tuerlinckx F, Gelman A, Vanpaemel W (2016). Increasing transparency through a multiverse analysis. Perspect. Psychol. Sci..

[47] Rosen WG (1980). Verbal fluency in aging and dementia. J. Clin. Exp. Neuropsychol..

[48] Tombaugh TN, Kozak J, Rees L (1999). Normative data stratified by age and education for two measures of verbal fluency: Fas and animal naming. Arch. Clin. Neuropsychol..

[49] Hills TT, Mata R, Wilke A, Samanez-Larkin GR (2013). Mechanisms of age-related decline in memory search across the adult life span. Dev. Psychol..

[50] Castro N, Siew CSQ (2020). Contributions of modern network science to the cognitive sciences: Revisiting research spirals of representation and process. Proc. Royal Soc. A: Math. Phys. Eng. Sci..

[51] Hoffman P (2018). An individual differences approach to semantic cognition: Divergent effects of age on representation, retrieval and selection. Sci. Rep..

[52] Ralph MAL, Jefferies E, Patterson K, Rogers TT (2017). The neural and computational bases of semantic cognition. Nat. Rev. Neurosci..

[53] Robinson L, Tang E, Taylor J-P (2015). Dementia: Timely diagnosis and early intervention. BMJ.

[54] Larson EB, Yaffe K, Langa KM (2013). New insights into the dementia epidemic. N. Engl. J. Med..

[55] Fillenbaum GG, van Belle G, Morris JC, Mohs RC, Mirra SS, Davis PC, Tariot PN, Silverman JM, Clark CM, Welsh-Bohmer KA (2008). Consortium to establish a registry for alzheimer’s disease (cerad): The first twenty years. Alzheimer’s & Dementia.

[56] Newman ME (2006). Modularity and community structure in networks. Proc. Natl. Acad. Sci..

[57] Barrat A, Barthelemy M, Pastor-Satorras R, Vespignani A (2004). The architecture of complex weighted networks. Proc. Natl. Acad. Sci..

[58] Latora V, Marchiori M (2001). Efficient behavior of small-world networks. Phys. Rev. Lett..

