# Supplementary material for: Structural differences in the semantic networks of younger and older adults
Source: Sci Rep. 2022 Dec 12;12:21459. doi: 10.1038/s41598-022-11698-4 (PMC9744829; doi:10.1038/s41598-022-11698-4)
Supplement: Supplementary file 1 — Supplementary Information. [file 41598_2022_11698_MOESM1_ESM.pdf]

Supplementary Material

Methods

Fluency data sets

The first fluency data set combines two previously published longitudinal data sets. One data set was obtained from Hills, Mata, Wilke, & Samanez-Larkin (2013) and contains three waves of responses to a one-minute animal fluency task. At time point one the data included a total of 201 participants aged 27 to 99 (Mdn = 68). For our analyses we used the data of the first wave, to avoid any practice effects and problems associated with participant attrition. The other half was obtained from the Midlife in the United States (MIDUS) longitudinal study. In the context of the MIDUS3 study, one-minute animal fluency data were recorded over the phone from 104 individuals aged 34 to 83 (Mdn = 59). To render these data machine-readable, we transcribed the audio recordings (see section on Fluency preprocessing). We excluded from both data sets in total 21 individuals who had a mini-mental state value lower than 26 and produced fewer than 10 items, leaving 284 participants for analysis. We created groups of younger and older adults by splitting the data at the median age . This resulted in two groups of 142 individuals aged 29 to 65 years old and 66 to 94 years old, respectively.

The second and third fluency data sets stem from our study 1, which was collected in the context of another study on age-differences in decision making run at the Max Planck Institute (MPI) for Human Development, Berlin. We collected 10-minute fluency data for both animals and countries from 71 older adults and 41 younger adults. Responses were recorded using a microphone and transcribed by us (see section on Fluency preprocessing). Along with the audio recordings, we obtained data from the cognitive battery typically included in aging at the MPI for Human Development. This battery included measures of working memory span (OSPAN; Unsworth, Heitz, Schrock, & Engle, 2005), vocabulary size

(Lehrl, Triebig, & Fischer, 1995), numeracy (BNT; Cokely et al., 2012), maximization in decision making (Schwartz et al., 2002), the big five personality traits (Borkenau & Ostendorf, 2008), memory controllability (MCI; Lachman, Bandura, Weaver, & Elliott, 1995), associative recall (Shing et al., 2010), quality of life (SF-12; Ware, Kolinski, & Keller, 1995), depression (GDS; Yesavage et al., 1983), positive and negative affect (PANAS; Watson, Clark, & Tellegen, 1988), and mental status (MMSE; Folstein, Folstein, & McHugh, 1975). Participants were recruited through the internal participant database of the MPI of Human Development. The older adults' group ranged from 65 to 80 years with a median age of 70 years, the younger adults' age ranged from 17 to 33 with a median age of 25 years. Participants were paid 10€/hour for participation in this study and the study lasted roughly two hours.

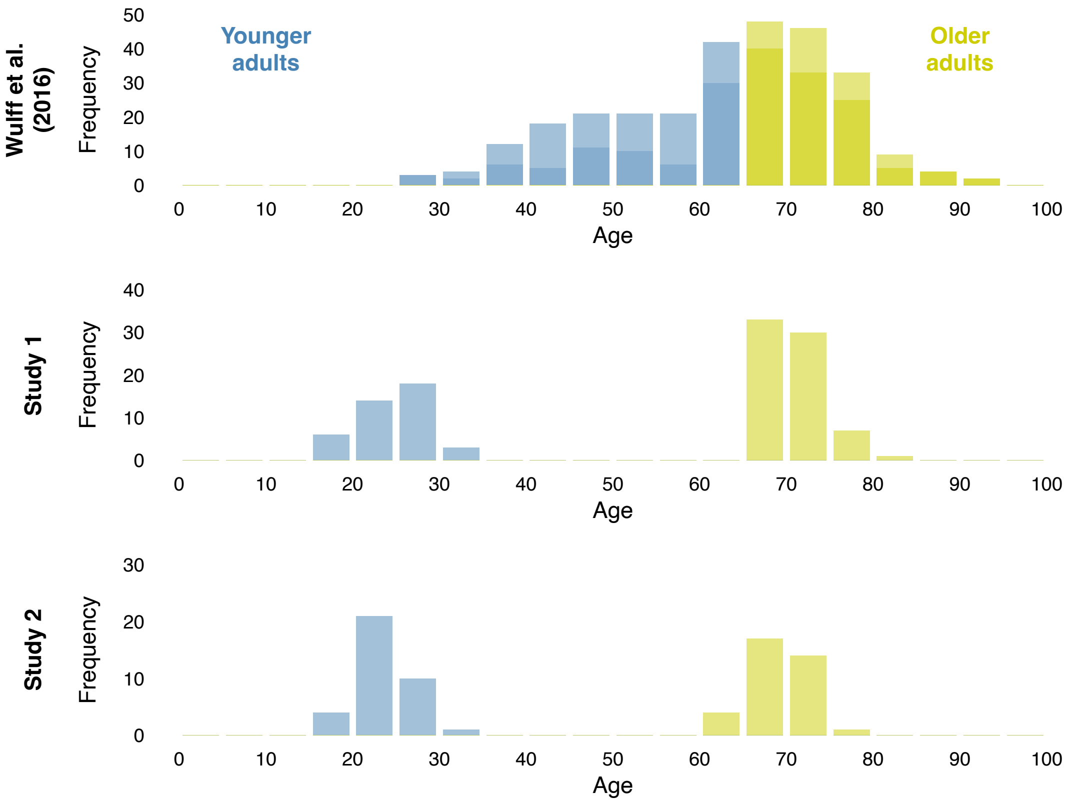

Figure S1. Age distributions in the data of Wulff et al. (2016), study 1, and study 2. The darker and lighter regions in the top panel represent the Hills et al. (darker) and MIDUS (lighter) portions of the data set.

The fourth fluency data set stems from our study 2 and was collected at the Max Planck Institute for Human Development using participants from the MPI's internal database. We recorded via microphone responses to a 10-minute animal fluency task and transcribed the responses (see section on Fluency preprocessing). We also collected responses to the cognitive aging battery typically employed at the MPI.

Study 1, 2 and 3 were approved by the internal review board of the Max Planck Institute for Human Development.

Table S1
Characteristics of the Fluency Data sets Included in the Analysis

Study	Younger adults	Older adults	Main variables	Additional variables
1	N = 142<br>age 29-65<br>70% female	N = 142<br>Age 66-94<br>70% female	Animal fluency (1min)	-
2	N = 41<br>age 18-34<br>48% female	N = 71<br>age 66-81<br>41% female	Animal fluency (10min)<br>Country fluency (10min)	PANAS, Vocabulary, BNT, Schwartz maximization, Big Five, MCI, Associative recall, OSPAN, GDS
3	N = 36<br>age 18-32<br>42% female	N = 36<br>age 65-78<br>63% female	Animal fluency (10min)	Same as study 2

Fluency Preprocessing

Fluency responses available as audio files were, first, transcribed using the Penn TotalRecall annotation software (http://memory.psych.upenn.edu/TotalRecall). In the next step, responses were scrutinized for category membership and spelling. For category

membership, we used a lenient criterion to retain as much of the original data as possible. In the case of animals, all nonfictional entries that described entire, nonhuman animals were accepted. This led us to exclude a few cases from the data, such as Godzilla, cat eye, or animal trainer. Similarly, in the case of countries, we accepted all existing and named territories such as Istrien, a region of Italy, Croatia *and* Slovenia, the desert Sahara or cities, but not nonexistent, fictional territories such as Middle-earth. Spelling was hand-corrected on the basis of the Merriam-Webster online dictionary. Overall 96.8% to 99% of responses were retained in the analysis. For details see table S2.

Table S2

Fluency Processing Statistics

Data sets	*N* productions		unique/N[a]		% duplicate		% synonyms		% removed	
	YA	OA	YA	OA	YA	OA	YA	OA	YA	OA
S1 - Animals	22	18.6	.096	.112	7.3	7.9	1.2	1.5	1	1.9
S2 - Animals	93.1	101.8	.148[b]	.178[b]	3.2	10.8	2.7	3.3	.8	.9
S2 - Countries	77.6	80.3	.083[b]	.108[b]	4.2	12.6	10.8	9.9	.5	.6
S3 - Animals	98.1	97.5	.174	.188	5.8	9.6	3.7	4.4	2.2	3.2

Legend

[a] Ignoring duplicate productions.

[b] Based on repeated random samples of 30 individuals per group.

Network Inference from Fluency Data

Networks were inferred from semantic fluency based on the community model devised by Goñi and colleagues (2010) and studied by Zemla and Austerweil (2018). The model encompasses the following two-step procedure. First, nodes and edges are included for every pair of responses that occurred within a distance of *l* responses. For instance, for the response sequence "dog, cat, mouse, rabbit" and a criterion of *l* = 2, edges would be included for all pairs less than three responses apart excluding only the pair dog and rabbit, which are three responses apart. Second, an edge is identified as a true edge if the frequency of the connected words occurring within *l* or fewer steps apart exceeded a frequency threshold t_{min}

(the absolute minimum required frequency), as well as a frequency threshold t_{chance}. The latter was derived from the probability p_{ij}^{linked} of those words occurring within *l* responses by chance with p_{ij}^{linked} calculated as

$$p_{ij}^{linked} = p_{ij}^{co-occur} * p_{ij}^{\leq l}$$

where $p_{ij}^{co-occur}$, the probability of two words to co-occur within a fluency sequence, and $p_{ij}^{\leq l}$, the probability that two responses are no more than *l* responses apart, being calculated as

$$p_{ij}^{co-occur} = \frac{f_i f_j}{MM}$$

and

$$p_{ij}^{\geq l} = \frac{2}{N(N-1)}\left(lN - \frac{l(l+1)}{2}\right)$$

with f_i, f_j denoting the number of times two responses occur across *M* sequence and *N* denotes the average number of productions per sequence. t_{chance} is then defined as the $1-\alpha$ quantile of the binomial distribution $B\left(M, p_{ij}^{linked}\right)$. The model thus encompasses three parameters: the window size *l*, the minimum threshold t_{min}, and probability α to determine the chance-threshold t_{chance}. Goñi et al. (2010) and Zemla and Austerweil (2018) found parameters to $l = 2$, $t_{min} = 1$, and $\alpha = .05$ to produce plausible networks that predicted human similarity judgments better than six other available methods (Zemla & Austerweil, 2018).

Macroscopic Structure of Inferred Fluency Networks and Multiverse Analysis

Using our network inference method, we inferred networks for younger and older adults for each of the four data sets. For the two data sets of study 2 this implied equating the groups of younger and older adults by means of bootstrap analyses. That is, the 41 younger adults were compared to random draws of 41 individuals from the older adults‘ group. To

avoid confounding influences of network size, the results were determined based on the giant component of the common subgraph of both groups. All estimates were derived on the basis of 1,000 bootstrap samples.

Table S3 shows the numeric results for standard settings of $l = 2$, $t_{min} = 1$, and $\alpha = .05$ including group differences and associated confidence intervals. These show systematic group differences for two of the three characteristics considered. Specifically, younger adults showed higher densities (k) and lower average shortest path lengths (L) for each of the four data sets. Only the clustering coefficient showed a mixed pattern with one data set showing larger values for younger adults and three showing smaller ones as compared to older adults.

Table S3

Macroscopic Structure of Fluency-based Semantic Networks of Younger and Older Adults based on 1,000 Bootstrap Samples

			Network		
	Age	|N|	<k>	C	L
Study 1	YA	104	2.87	.32	3.05
	OA	104	2.47	.31	3.52
	Δ [a]	0 [b]	0.39	.01	-.46
	CI [c]	-	(-.13, .92)	(-.04, .06)	(-.64, -.28)
Study 2 - Animals	YA	233.7	5.02	.30	2.82
	OA	233.7 [d]	3.90 [d]	.31 [d]	3.16 [d]
	Δ	0	1.13	-.01	-.35
	CI	-	(.21, 2.04)	(-.59, .03)	(-.65, -.04)
Study 2 – Countries	YA	158.8	5.38	.31	2.61
	OA	158.8 [d]	4.61 [d]	.35 [d]	2.82 [d]
	Δ	0	.77	-.04	-.21
	CI	-	(-.28, 1.81)	(-.09, .02)	(-.49, .07)
Study 3	18-32	178	2.89	.31	3.48
	65-78	178	2.55	.33	4.33
	Δ	0	.36	-.03	-.84

CI	-	(-.56, 1.28)	(-.07, .02)	(-1.19, -.5)

Legend
[a] Difference between younger and older adults. [b] Network sizes were equated to across age groups. [c] Confidence interval. [b] results based on 41 randomly drawn sequences of older adults to match the 41 sequences of younger adults.

To evaluate the robustness of these results across different, possibly equally justifiable implementations of our inference method, we ran a multiverse analysis (Steegen, Turlinckx, Gelman, Vanpaemel, 2016) with a total of 27 parameter combinations. Specifically, we let $l = [1, 2, 3]$, $t_{min} = [0, 1, 2]$, and $\alpha = [.01, .1, 1]$. The multiverse analysis presented in Figure S1 corroborates the systematic differences found for the average degree k and the average shortest path length L. Specifically, we observed 96% and 97% of all implementations to produce results for k and L, respectively, that were consistent with those presented in Table S1 and Figure S2. However, for the average clustering coefficient C, results were found to be much more inconsistent with only 62% agreeing with the majority pattern in Table 1 of larger clustering for older adults as compared to younger adults. Conversely, 38% of implementations indicated larger clustering for the opposite pattern, smaller clustering for older as compared to younger adults. These analyses indicate that we should place less confidence in the observed group differences for clustering as compared to those observed for degree and shortest path length.

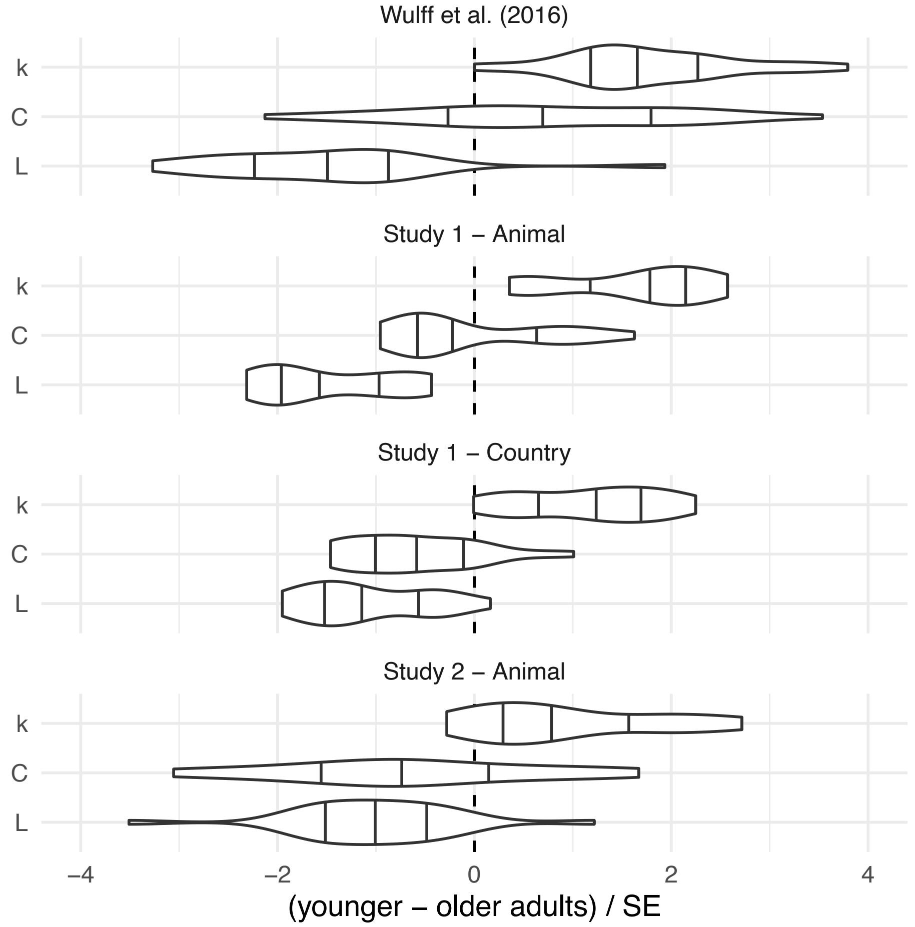

Figure S2. Multiverse analysis. Figures shows the comparison of younger and older adults' inferred macroscopic networks characteristics across 27 implementations of our inference method for each of the four data sets. Violins illustrate the distribution of the differences between younger and older adults divided by the bootstrap standard error for the 27 implementations on the basis of 1,000 bootstrap samples. Vertical lines indicate quartile boundaries.

Similarity Ratings Using Tablets

A total of 36 younger and 36 older adults participated in the study. The older adults' age ranged from 65 to 78 years with a median age of 70 years, the younger adults' age ranged from 18 to 32 with a median age of 23.5. Groups were matched in terms of education.

Participants were paid 10€/hour for participation in the lab session, which lasted roughly 2 hours, and a flat fee of 10€ for providing the similarity ratings.

Participants in our study provided similarity ratings to a total of 1,953 unique pairs of animals. The set of pairs was created based on the fluency responses from study 2. Specifically, we selected a list of 66 animals that were retrieved by at least 33% of younger and older adults. From these we eliminated the words *fish*, *bear*, and *insects* to avoid category-token judgments, leaving 63 animals words and (63*62)/2 = 1953 possible word pairs for similarity judgments. These 63 words covered 41% and 41.3% of all responses of younger and older adults, respectively. We added a set of 315 word pairs sampled evenly from the main set to estimate the reliability of similarity ratings.

Participants provided similarity ratings via a Google Nexus Tablet that they took home after attending the first lab session including the semantic fluency task and the cognitive battery. Participants provided similarity ratings on a scale from 1 to 20. Instructions on the similarity ratings were minimal so as not to influence individuals in any particular way. Participants were asked to conduct sessions of 30 minutes in the morning and the evening of every day during the study. On average, younger adults completed the study in 8.9 days, older adults in 6.9 days. The responses to the reliability set revealed that individuals were highly reliable in their similarity ratings. Specifically, younger and older adults showed average correlations of r = .76 and r = .74, respectively, which we found not to be affected whether or not the pair of animals was presented in the same order (i.e., bear-mouse vs. mouse-bear).

In contrast to our approach to derive networks using fluency data, the similarity rating data have three major advantages. First, they permit the construction of weighted networks, in which edges can represent the strength of similarity between two animals. Second, it is not necessary to induce common subgraphs as all networks will by design contain all animals as

nodes. Third, they permit the construction of networks on the level of the individual, permitting us to evaluate differences between younger and adults with respect to within-group individual differences and, thus, to draw inferences using standard statistical procedures. To derive individual-level networks from similarity ratings, we first mapped individuals' similarity minimum and maximum ratings to the range of 0 and 1. This was necessary to account for the fact that individuals appear to have used the rating scale slightly differently, with rating spans (max minus max rating) ranging from 20 to as low as 14.

After ratings had been normalized we created five networks for each individual that included edges for animal pairs with similarity larger than 0, .1, .2, .3, and .4, respectively. Eliminating some subset of edges was necessary in order to determine the clustering coefficient and using multiple criteria allowed us to assess the robustness of our approach. We characterized each individual's macroscopic network structure using the average degree *k*, i.e., the number of neighbors of a node, the average strength, *s*, i.e., the average edge weights to a node's neighbors, the average shortest path length and the average clustering coefficient. For clustering we used the following formula for for weighted networks (Barrat, Barthélemy, Pastor-Satorras, & Vespignani, 2004):

$$C^w = \frac{1}{N}\sum_i c_i^w = \frac{1}{N}\sum_i \frac{1}{s_i(k_i - 1)}\sum_{j,h} \frac{(w_{ij} + w_{ik})}{2}$$

with w_{ij} being the weight of the edge between nodes i and j, s_i being strength of node i, defined as the sum of edge weights over all of its neighbors j, k_i being degree of node i, and N being the number of nodes in the network.

The results presented in Table S4 show that older and younger adults differed systematically with regard to all four network characteristics. Younger adults showed across all threshold levels larger connectivity in terms of node degree and strength, larger clustering coefficients, and shorter average shortest path lengths. Confidence intervals indicate at least two reliable effects per structural property, mainly for the three lower thresholds. For higher

thresholds, the differences seem to be overall small suggesting that age differences manifested primarily in remote regions in the networks. Furthermore, with larger thresholds networks effectively shrink in size due to node islands, resulting in more volatile results.

Table S4

Comparison of Weighted Networks Based on Similarity Ratings Across Five Threshold Levels

Threshold	Group	k	s	C	L
0	Younger adults	50.7 (11.8)	16.0 (5.89)	.870 (.135)	.588 (.179)
	Older adults	37.0 (17.1)	12.5 (7.61)	.750 (.191)	.732 (.375)
	Difference	13.7	3.5	.121	-.146
	CI	(7, 20.5)	(.4, 6.5)	(.045, .196)	(-.281, -.010)
.1	Younger adults	42.4 (14.4)	15.5 (6.12)	.773 (.163)	.603 (.208)
	Older adults	29.0 (17.1)	12.0 (7.67)	.676 (.183)	.774 (.443)
	Difference	13.3	3.4	.097	-.174,
	CI	(6.2, 20.5)	(.3, 6.6)	(.018, .176)	(-.332, -.017)
.2	Younger adults	28.5 (13.0)	13.6 (6.05)	.636 (.128)	.648 (.312)
	Older adults	21.2 (14.2)	11.0 (7.35)	.587 (1.68)	.804 (.476)
	Difference	7.2	2.6	.050	-.154
	CI	(1, 13.4)	(-.5, 5.6)	(-.018, .118)	(-.336, .027)
.3	Younger adults	20.1 (9.88)	11.5 (5.38)	.571 (.115)	.679 (.312)
	Older adults	16.1 (10.5)	9.7 (6.65)	.528 (.161)	.772 (.373)
	Difference	4.1	1.8	.044	-.080
	CI	(-.6, 8.7)	(-.9, 4.5)	(-.020, .108)	(-.249, .090)
.4	Younger adults	14.6 (6.72)	9.58 (4.43)	.543 (.113)	.672 (.320)
	Older adults	12.6 (8.78)	8.47 (6.18)	.496 (.173)	.777 (.274)
	Difference	1.9	1.1	.048	-.093
	CI	(-1.6, 5.5)	(-1.4, 3.5)	(-.019, .115)	(-.248, .062)

Controlling for Education and Gender

In our assessment of similarity rating networks, younger and older adults were not perfectly matched in terms of gender and education level, which may have possibly confounded the effect of age group. To test whether difference in education and gender between the age groups may have contributed to the structural differences presented in the main analyses, we ran separate regressions predicting each network property using age group,

education level (college level education yes/no), and gender as predictors in linear regression separately for each edge similarity cutoff value in [0, .1, .2, .3, .4]|. Figure S3 shows the estimates and associated 95% confidence intervals. The results show that age group still has substantial effects on each of the network properties for small cutoff values despite controlling for education and gender. They also show noticeable effects of education on most network properties, but no effects of gender. These analyses demonstrate that the effects of age group on network structure are not driven by group differences education and gender.

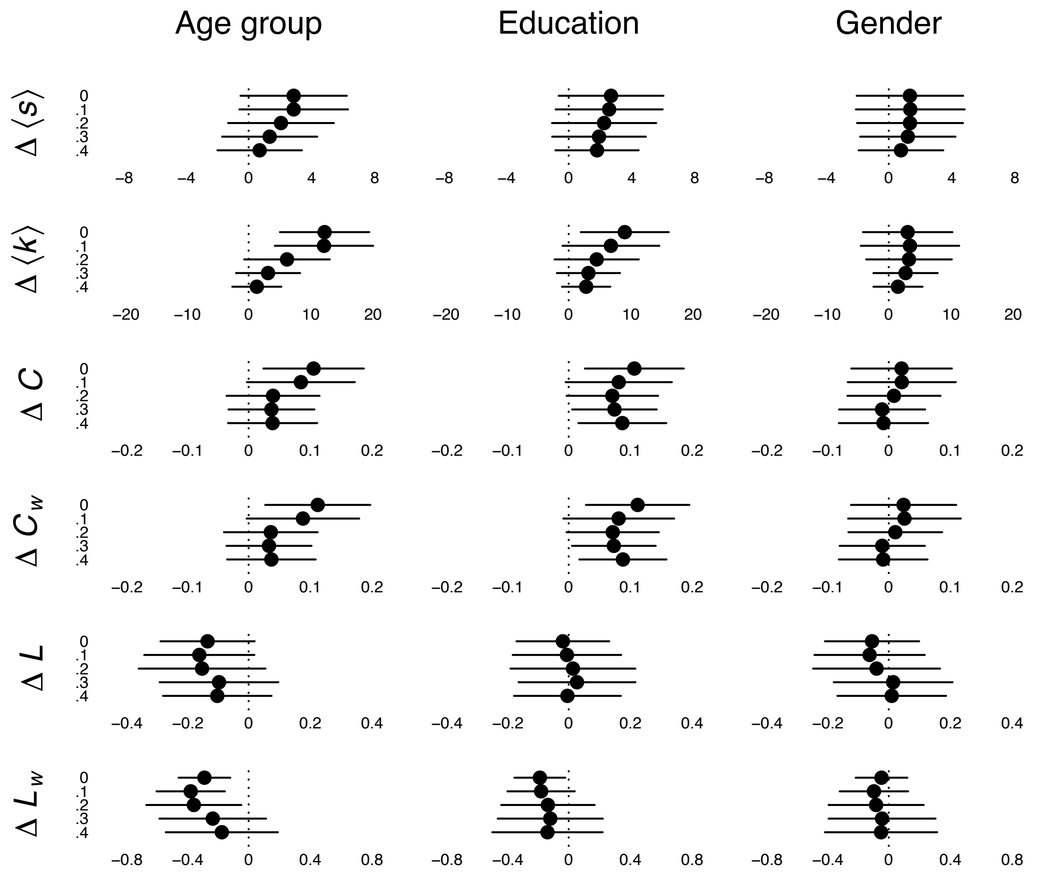

Figure S3. Effects of age group, education, and gender on network structure. Points and horizontal lines reflect the regression estimates and associated 95% confidence intervals for models predicting network structure using age group, education, and gender separately for each of the three characteristics—degree, clustering, and shortest path length—in weighted

and unweighted networks and the five different cutoff values [0, .1, .2, .3, .4, .5] for edge similarity.

Individual Versus Aggregate Networks

The individual-level similarity rating data provide an opportunity for assessing the effect of aggregation on the comparison of younger and older adult network structures. We calculated group-wise aggregate networks by averaging the edge weights of all younger and older adults, respectively. Figure S4 shows the network characteristics of the resulting aggregate networks compared to the characteristics observed for individuals. We found that group-wise aggregate networks overestimate the network characteristics of both younger and older individuals. On average, the aggregate characteristics were higher than those of 69.7% of individuals. The overestimation was most pronounced for the clustering coefficient (81.9%), followed by the average shorted path length (75%) and degree (70.8%). Only for strength (51.4%) were individuals accurately represented by the aggregate network. Notably, the aggregate networks still reflected the group differences observed based on the individual networks. Thus, although aggregate networks were biased towards larger degrees, clustering coefficients, and average shortest path lengths, they seemed to be biased in roughly equal amounts for the two groups, retaining their relative position to one another.

The relatively benign aggregation bias may result from the fact that in this case all networks include, by design, the same set of nodes. When analyses aggregate not only across the presence and absence of edges, as in this case, but also across the presence and absence of nodes, as in the case of verbal fluence or free association networks, then aggregation biases will likely be more severe.

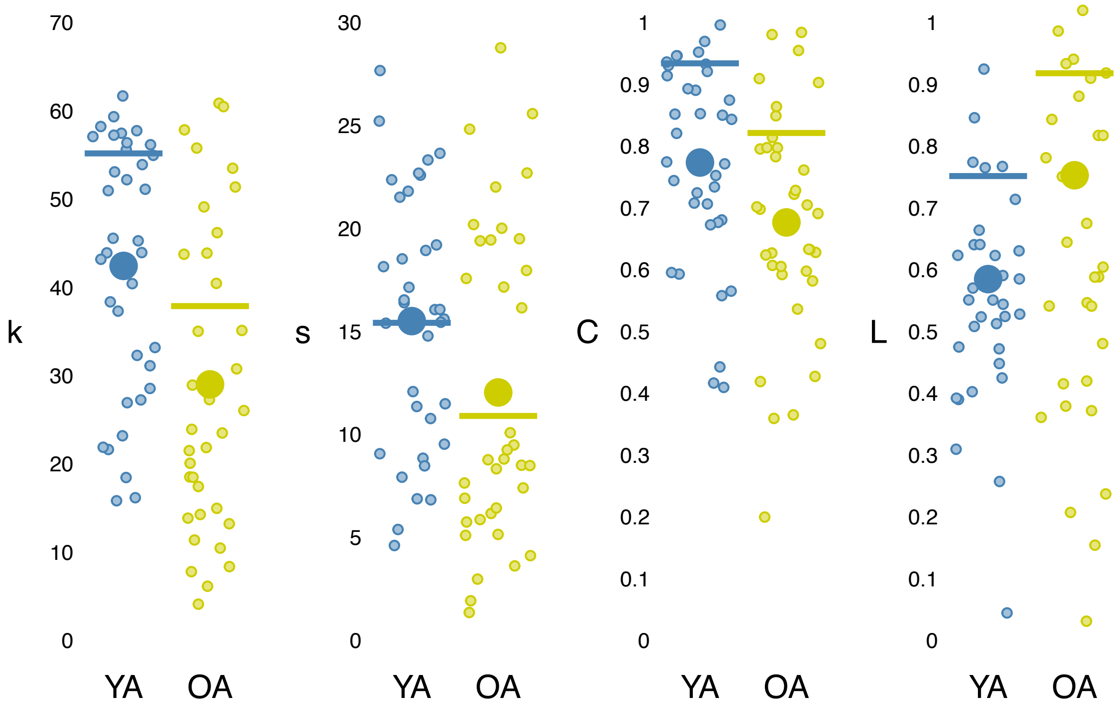

Figure S4. The effect of aggregation. The figure shows the network characteristics under w_{min} = .1 for each individual (small, open circles) and the respective group averages (large, solid circles). The horizontal bars show the same characteristics for group-wise, aggregate networks created by averaging the edge weights of individual networks separately for younger and older adults.

References

Abbott, J. T., Austerweil, J. L., & Griffiths, T. L. (2015). Random walks on semantic networks can resemble optimal foraging. *Psychological Review*, *122*(3), 558.

Barabási, A. L., & Albert, R. (1999). Emergence of scaling in random networks. *Science*, *286*(5439), 509-512.

Borkenau, P., & Ostendorf, F. (2008). NEO-FFI: NEO-Fünf-Faktoren-Inventar nach Costa und McCrae, Manual.

Cokely, E. T., Galesic, M., Schulz, E., Ghazal, S., & Garcia-Retamero, R. (2012). Measuring risk literacy: The Berlin numeracy test. *Judgment and Decision Making*.

Goñi, J., Arrondo, G., Sepulcre, J., Martincorena, I., de Mendizábal, N. V., Corominas-Murtra, B., ... & Villoslada, P. (2011). The semantic organization of the animal category: evidence from semantic verbal fluency and network theory. *Cognitive Processing*, *12*(2), 183-196.

Hills, T. T., Mata, R., Wilke, A., & Samanez-Larkin, G. R. (2013). Mechanisms of age-related decline in memory search across the adult life span. *Developmental Psychology*, *49*(12), 2396.

Hills, T. T., Jones, M. N., & Todd, P. M. (2012). Optimal foraging in semantic memory. *Psychological Review*, *119*(2), 431.

Holme, P., & Kim, B. J. (2002). Growing scale-free networks with tunable clustering. *Physical Review E*, *65*(2), 026107.

Lachman, M. E., Bandura, M., Weaver, S. L., & Elliott, E. (1995). Assessing memory control beliefs: The memory controllability inventory. *Aging, Neuropsychology, and Cognition*, *2*(1), 67-84.

Lehrl, S., Triebig, G., & Fischer, B. (1995). Multiple choice vocabulary test MWT as a valid and short test to estimate premorbid intelligence. *Acta Neurologica Scandinavica*, *91*(5), 335-345.

Schwartz, B., Ward, A., Monterosso, J., Lyubomirsky, S., White, K., & Lehman, D. R. (2002). Maximizing versus satisficing: Happiness is a matter of choice. *Journal of Personality and Social Psychology*, *83*(5), 1178.

Shing, Y. L., Werkle-Bergner, M., Brehmer, Y., Müller, V., Li, S. C., & Lindenberger, U. (2010). Episodic memory across the lifespan: The contributions of associative and strategic components. *Neuroscience & Biobehavioral Reviews*, *34*(7), 1080-1091.

Steegen, S., Tuerlinckx, F., Gelman, A., & Vanpaemel, W. (2016). Increasing transparency through a multiverse analysis. *Perspectives on Psychological Science*, *11*(5), 702-712

Steyvers, M., & Tenenbaum, J. B. (2005). The large-scale structure of semantic networks: Statistical analyses and a model of semantic growth. *Cognitive Science*, *29*(1), 41-78.

Ware, J. E., Kolinski, M., & Keller, S. D. (1995). *How to score the SF-12 physical and mental health summaries: a user's manual*. Boston: The Health Institute, New England Medical Centre, Boston, MA.

Unsworth, N., Heitz, R. P., Schrock, J. C., & Engle, R. W. (2005). An automated version of the operation span task. *Behavior Research Methods*, *37*(3), 498-505.

Yesavage, J. A., & Sheikh, J. I. (1986). 9/Geriatric depression scale (GDS) recent evidence and development of a shorter version. *Clinical Gerontologist*, *5*(1-2), 165-173.

Watson, D., Clark, L. A., & Tellegen, A. (1988). Development and validation of brief measures of positive and negative affect: the PANAS scales. *Journal of Personality and Social Psychology*, *54*(6), 1063.

Folstein, M. F., Folstein, S. E., & McHugh, P. R. (1975). “Mini-mental state”: a practical method for grading the cognitive state of patients for the clinician. *Journal of Psychiatric Research*, *12*(3), 189-198.

Zemla, J. C., & Austerweil, J. L. (2018). Estimating semantic networks of groups and individuals from fluency data. *Computational Brain & Behavior*, *1*(1), 36-58.
